# Perception of force and stiffness in the presence of low-frequency haptic noise

**DOI:** 10.1371/journal.pone.0178605

**Published:** 2017-06-02

**Authors:** Netta Gurari, Allison M. Okamura, Katherine J. Kuchenbecker

**Affiliations:** 1 Department of Physical Therapy and Human Movement Sciences, Northwestern University, Chicago, IL, United States of America; 2 Department of Mechanical Engineering, Stanford University, Stanford, CA, United States of America; 3 Haptic Intelligence Department, Max Planck Institute for Intelligent Systems, Stuttgart, Germany, and Department of Mechanical Engineering and Applied Mechanics, University of Pennsylvania, Philadelphia, PA, United States of America; University of Exeter, UNITED KINGDOM

## Abstract

**Objective:**

This work lays the foundation for future research on quantitative modeling of human stiffness perception. Our goal was to develop a method by which a human’s ability to perceive suprathreshold haptic force stimuli and haptic stiffness stimuli can be affected by adding haptic noise.

**Methods:**

Five human participants performed a same-different task with a one-degree-of-freedom force-feedback device. Participants used the right index finger to actively interact with variations of force (∼5 and ∼8 N) and stiffness (∼290 N/m) stimuli that included one of four scaled amounts of haptically rendered noise (*None*, *Low*, *Medium*, *High*). The haptic noise was zero-mean Gaussian white noise that was low-pass filtered with a 2 Hz cut-off frequency; the resulting low-frequency signal was added to the force rendered while the participant interacted with the force and stiffness stimuli.

**Results:**

We found that the precision with which participants could identify the magnitude of both the force and stiffness stimuli was affected by the magnitude of the low-frequency haptically rendered noise added to the haptic stimulus, as well as the magnitude of the haptic stimulus itself. The Weber fraction strongly correlated with the standard deviation of the low-frequency haptic noise with a Pearson product-moment correlation coefficient of *ρ* > 0.83. The mean standard deviation of the low-frequency haptic noise in the haptic stimuli ranged from 0.184 N to 1.111 N across the four haptically rendered noise levels, and the corresponding mean Weber fractions spanned between 0.042 and 0.101.

**Conclusions:**

The human ability to perceive both suprathreshold haptic force and stiffness stimuli degrades in the presence of added low-frequency haptic noise. Future work can use the reported methods to investigate how force perception and stiffness perception may relate, with possible applications in haptic watermarking and in the assessment of the functionality of peripheral pathways in individuals with haptic impairments.

## Introduction

Humans evaluate stiffness for a wide variety of tasks, such as determining the air pressure in a tire and assessing the ripeness of a piece of fruit. In each of these scenarios, the object is actively manipulated, and its surface displaces in response to the applied force. The amount that the surface deflects in response to the applied force depends on the object’s physical properties, with stiffer objects deflecting less than softer objects under the same magnitude of applied force. Prior work has used a variety of experimental protocols to show that the processes by which humans perceive force, displacement, and stiffness are relatable [1–7]. Even so, we do not fully understand how force perception relates to stiffness perception.

Humans perceive force and stiffness stimuli by integrating information from both kinesthetic and cutaneous mechanoreceptors [8–10]. Kinesthetic cues are defined as information relayed from mechanoreceptors that respond to mechanical stimulation of the body’s internal structures, including muscle spindles, Golgi tendon organs, and joint capsule receptors [11, 12]. Cutaneous cues are defined as information relayed from mechanoreceptors that respond to mechanical stimulation of the skin, specifically the slowly adapting (SA) type I, SA II, fast adapting (FA) I, and FA II units [13]. The Golgi tendon organs and SA I units are sensitive to low-frequency force stimulation and are known to contribute to human perception of both force and stiffness stimuli [8–10, 14]. Golgi tendon organs are thought to signal dynamic force information to the central nervous system and to combine with discharges from other types of receptors, including cutaneous mechanoreceptors, to create one’s estimate of static force [15, 16]. SA I units are densely located on the volar portion of the index finger [13] and are sensitive to sinusoidal haptic stimuli with frequencies <8 Hz [17].

The goal of this work is to identify haptic noise that, when added to suprathreshold haptic force stimuli and haptic stiffness stimuli, impacts the human ability to perceive both of these stimuli. Prior work demonstrates that mechanically stimulating an individual’s mechanoreceptors using haptic noise may affect human perception in numerous ways, including creating sensory illusions and enhancing, deteriorating, shifting, or having no impact on the individual’s ability to identify a haptic stimulus [18–25]. For example, humans are less likely to detect a near-threshold force stimulus at the finger pad when suprathreshold low-frequency Gaussian force noise is added to the force stimulus [19]. These studies, however, have not demonstrated how haptic noise that affects human perception of force stimuli affects human perception of stiffness stimuli. In this work, we identified additive low-frequency haptic noise that affected the human ability to perceive a haptic force stimulus and a haptic stiffness stimulus.

The manuscript is organized as follows. First, we provide general information about the experimental methods used to evaluate the ability of participants to precisely identify the magnitude of force and stiffness stimuli. Then, we discuss the controllers that rendered these force and stiffness stimuli on a force-feedback device and describe a new method for evaluating the quality of these haptic stimuli. Next, we describe the analyses characterizing the perceptual performance of the participants and show that adding low-frequency haptically rendered noise to haptic stimuli degrades the quality of the rendered haptic stimuli, as well as the precision with which participants assess the magnitude of the haptic force and stiffness. These findings demonstrate that the human ability to perceive both suprathreshold haptic force stimuli and stiffness stimuli is altered by the magnitude of the low-frequency haptic noise present in the signals.

## Experimental methods

The methods presented here are a subset of a larger body of work that aims to model how humans perceive stiffness [26]; we discuss the experimental setup and procedures relevant to characterizing human perception of haptic force and stiffness stimuli in the presence of haptic noise.

### Participants

The Johns Hopkins University Homewood Institutional Review Board granted approval to conduct the study, and all participants provided written informed consent. Two males and three females who were healthy and right hand dominant with ages ranging between 20 and 33 participated. The participants reported no neurological illnesses or right hand impairments, and they were monetarily compensated.

### Experimental setup

**Fig 1:**
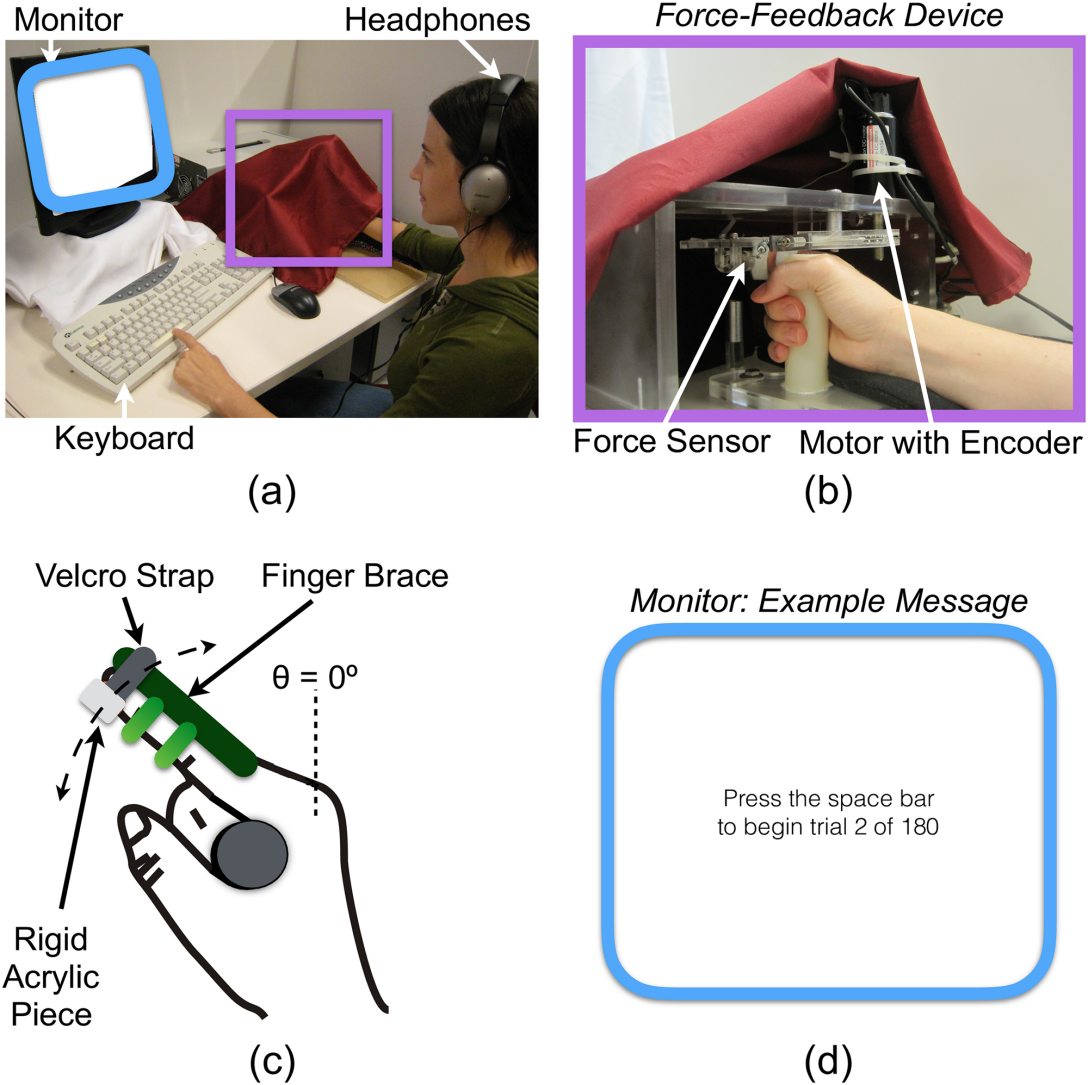
Experimental setup. (a) Experimental setup: The participant wears noise-canceling headphones that mask auditory distractions and provide experimental cues. The computer monitor displays trial-related commands to the user throughout the testing. The custom force-feedback device and the participant’s right hand are hidden by a red cloth during experimentation, and the participant presses keys with the left hand to provide trial-related feedback. (b) Force-feedback device: The participant rotates the right index finger about the metacarpophalangeal joint, and a motor with encoder applies a force to and measures the angular position of the participant’s finger. A force sensor measures interaction forces. (c) User interaction: The participant grasps a cylindrical tube with the right hand and presses on a rigid acrylic disk attached to the force-feedback device using the center of the right index finger’s distal finger pad. The participant wears a finger brace on the right index finger, and this finger is attached to the force-feedback device via a velcro strap that connects to the rigid acrylic piece. (d) Sample message: The monitor displays messages to instruct participants how to proceed throughout the trials. Images are adapted from Fig 1 of [27] © IEEE, and the participant shown in this figure provided written informed consent (as outlined in the PLOS consent form) to publish this photo.

The experimental setup is shown in Fig 1. Auditory and visual cues are masked by having the participant wear noise-canceling headphones that play white noise and by concealing the participant’s right hand and the force-feedback device with a cloth. The headphones also play sounds relevant to the experiment, and the monitor provides trial-related feedback. The participant responds to trial-related prompts by using the left hand to press keys on the keyboard.

The custom force-feedback device shown in Fig 1(b) generates the haptic stimuli. The participant interacts with the device by grasping the cylindrical tube with the right hand, inserting the index finger into the Velcro strap, and rotating the finger about the metacarpophalangeal (MCP) joint (Fig 1(c)). The participant wears a finger brace on the right index finger to prevent motion about the proximal and distal interphalangeal joints. The surface in contact with the finger pad is rigid acrylic, which requires the participant to rely on both cutaneous and kinesthetic mechanoreceptors to assess the stiffness of the haptic stimulus [8, 28].

A non-geared, backdrivable Maxon RE 40 DC motor (Sachseln, Switzerland) with a capstan drive ratio of 10:1 applies a force, *F*_cmd_, to the participant’s finger pad, and a HEDS 5540 encoder attached to the motor measures angular finger position, *θ*_meas_. An ATI Nano17 6-axis force/torque sensor (North Carolina, United States) located at the fingertip measures the interaction force, *F*_meas_. We direct readers to [29] for a more extensive description of the device.

### Task

**Fig 2:**
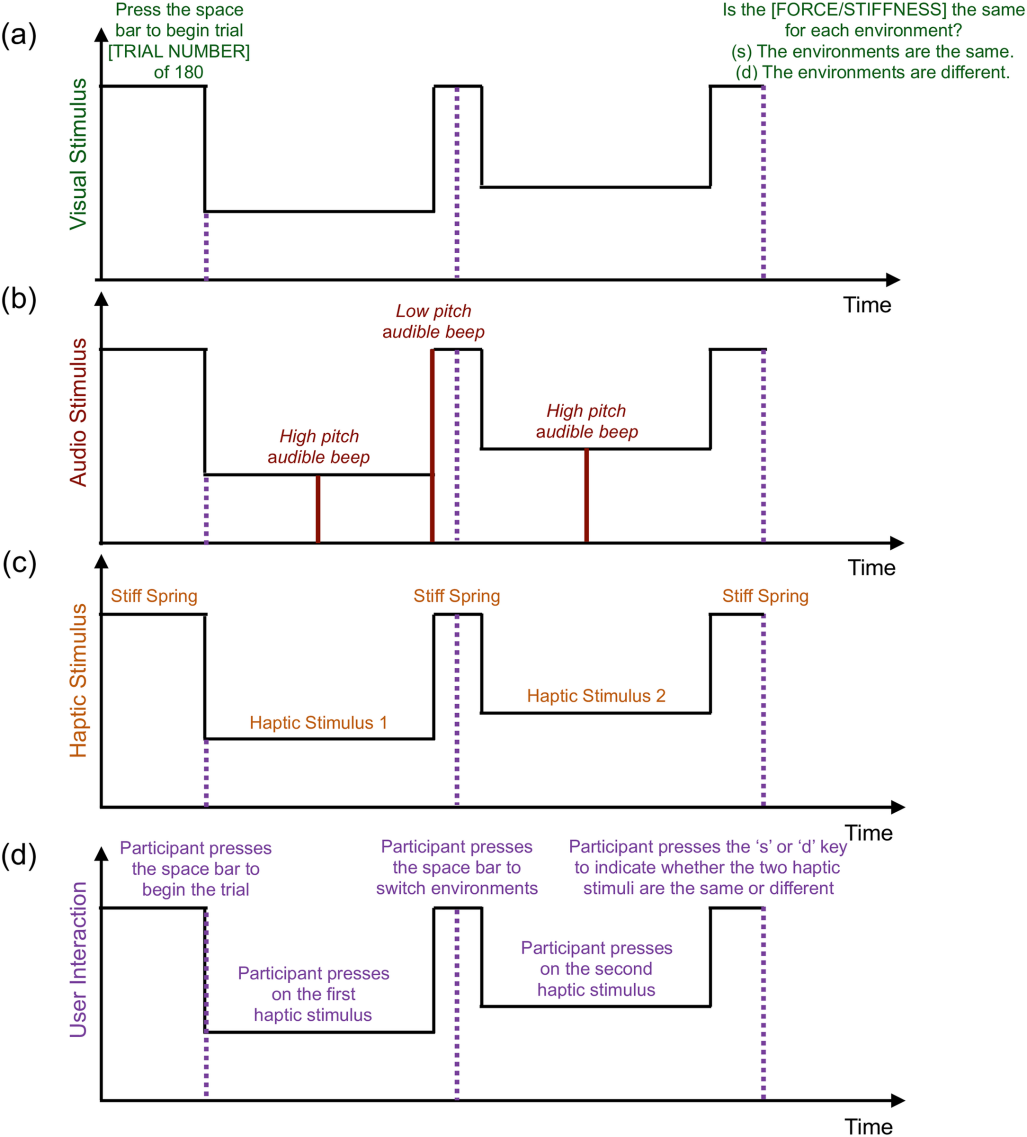
Trial timeline. Visual depiction of the (a) visual stimuli, (b) audio stimuli, (c) haptic stimuli, and (d) user interaction during the same-different task that a participant performs within a trial. The participant pushes the space bar with the left hand to begin the trial, then presses with the right index finger on the first haptic stimulus until an audible beep plays, and next returns the right index finger to the initial location. Following, the participant pushes the space bar with the left hand, waits 0.1 s, presses with the right index finger on the second haptic stimulus until an audible beep plays, and finally returns the right index finger to the initial location. Last, the participant responds whether the two haptic stimuli are the same by pushing the appropriate keyboard key with the left hand.

The same-different task that a participant performs for every trial is summarized in Fig 2. At the start of a trial, the participant’s right index finger is extended (*θ* = 0° in Fig 1(c)), and the device renders a stiff bilateral spring with a desired force of *F*_des_ = 4000 N/m · *x*_meas_ = 4000 N/m · *l*_*f*_ *θ*_meas_ to physically immobilize the finger; here, *l*_*f*_ is the measured length, in meters, from the center of the MCP joint to the center of the participant’s right index finger’s distal finger pad. The participant is instructed not to apply a force at this time. The participant then presses the space bar with the left hand to begin the trial, and the first haptic stimulus renders to the right index finger.

The participant moves the finger to a randomly commanded penetration arc length of P = 0.02, 0.025, 0.03, or 0.035 m (arc lengths were selected based on [7]). When the finger reaches the target arc length, an audible beep plays, and the participant rotates the finger back to *θ* = 0°, after which a lower-pitched beep plays. The stiff spring renders again, and the participant presses the space bar with the left hand to switch to the second haptic stimulus. The stiff spring renders for an additional 0.1 s to encourage the participant to wait until the second haptic stimulus renders prior to applying a force and rotating the right index finger. Then the participant rotates the right index finger to a new non-equidistant randomly commanded penetration arc length, the higher-pitched audible beep plays when the finger reaches the target arc length, and finally the participant rotates the finger back to *θ* = 0°. Last, the participant indicates whether the two haptic stimuli (i.e., the two Forces or the two Stiffnesses) are the same or different by pressing with the left hand on the ‘s’ or ‘d’ keyboard key, respectively. The commanded penetration arc lengths, P, were variable within and across trials to ensure that the participant discriminates between stiffness stimuli using stiffness cues rather than force cues [7].

### Data collection procedures

We employed a same-different task design based on the signal detection theory methods outlined in [30] to evaluate each participant’s force and stiffness perceptual capabilities. A benefit to the signal detection theory approach is that a human’s sensitivity and response bias can be independently measured, such that the human perceptual process is separated from the decision making process.

Each participant’s perceptual capabilities were evaluated across three stimulus types (Force A, Force B, Stiffness) at four haptically rendered noise scaling values (*None*, *Low*, *Medium*, *High*) for a total of twelve testing conditions. We tested two force stimuli with differing amplitudes (i.e., Force A, Force B) to investigate the impact of the signal-to-noise ratio on participant perception of force. The haptically rendered noise was designed as white Gaussian force noise (i.e., a random variable with a mean value of 0 N and a standard deviation of 1 N) that was low-pass filtered with a cut-off frequency of 2 Hz; this haptically rendered noise was added to the commanded forces when the haptic device rendered each force and stiffness stimulus. We characterized the ability of our participants to identify the magnitude of the force and stiffness stimuli in the absence and presence of this haptically rendered noise.

Data were collected across seven sessions, each session lasting less than 90 min to avoid participant fatigue and boredom. Haptically rendered noise was not added to the haptic stimulus for three conditions (i.e., noise level: *None*), while haptically rendered noise was added to the haptic stimulus for nine conditions (i.e., noise levels: *Low*, *Medium*, *High*). Each participant completed the three no-added-haptic-noise conditions across two to three consecutive days and the nine added-haptic-noise conditions within the span of no more than eight days. Presentation order of the testing conditions within each type was randomized for each participant. Two of the participants completed the noise level *None* conditions prior to the noise level *Low*, *Medium*, and *High* conditions, whereas the three remaining participants did the opposite.

For each of the twelve testing conditions, a participant completed six task-learning trials, 36 practice trials, and 144 testing trials. 36 practice trials were included to avoid the possibility of participants learning throughout the testing trials, and 144 testing trials were selected to obtain a decent resolution when characterizing each participant’s perceptual capabilities while avoiding boredom and fatigue. The participant completed all 186 trials for each condition consecutively in order to ensure that he or she was familiar with the task throughout all testing trials.

Each set of trials was counterbalanced across two trial types—standard-standard trials (which rendered two standard signals) and standard-comparison trials (which rendered one standard and one comparison signal). The magnitude of the haptically rendered noise remained constant within a condition—either *None*, *Low*, *Medium*, or *High*—whereas the magnitude of the haptic stimulus was set as either the standard signal or the comparison signal for each trial. During the standard-comparison trials, presentation of the comparison signal as the first or second stimulus was counterbalanced. Additionally, the commanded penetration arc lengths, P, were counterbalanced across all haptic stimuli and trials. We required the participant to press each haptic stimulus exactly once so that we could standardize their movements and therefore control the amount of information made available during each trial.

The participant learned how to perform the task for each condition during the task-learning trials. For these trials only, the comparison signal was set to be a much greater value than the standard signal so that, according to the experimenter’s pilot testing, the two signals were easily distinguishable from one another and the participant could focus on learning the task procedures. Upon completion of each task-learning trial, the experimenter indicated to the participant whether his or her response was correct. Throughout the trials the monitor visually depicted the speed of the participant’s finger by moving a short horizontal line up and down. The participant was instructed to maintain a speed that would keep the moving line within the range of two fixed horizontal lines at vertically offset locations corresponding to a minimum and maximum speed. The minimum speed of 10°/s ensured that the participant could feel a change in finger position [31], and the maximum speed of 80°/s avoided motions outside of the closed-loop bandwidth of the device (as discussed in the ‘*Controllers*’ section below).

Next, the participant completed 180 practice and testing trials under the same condition as the just completed task-learning trials; the participant was unaware that the first 36 trials were practice. A minimum break of 15 s was taken after every 16 trials, and additional/longer breaks were taken if desired. The comparison signals for these experimental trials were selected during pilot testing with the aim of ensuring that participants were not always guessing and were not always correct when discriminating between the standard stimulus and comparison stimulus for each combination of stimulus type and noise level. During the practice and testing trials, the experimenter did not indicate to the participant whether his or her response was correct, and visual speed indicators were not displayed to enforce that the participant focus on feeling the haptic stimuli.

## Haptic stimuli

Below we indicate the magnitude of the force and stiffness stimuli that were haptically rendered (i.e., standard and comparison force or stiffness signals), we describe the controllers that created the haptic stimuli, and we discuss the analyses developed to characterize the quality of the haptically rendered stimuli.

### Magnitude of haptic stimuli

This study identifies the precision with which a participant can perceive a standard signal, *μ*_*S*,*α*_, corresponding to a Force A of *μ*_*S*,*F*_*A*__ ≈ 5 N, Force B of *μ*_*S*,*F*_*B*__ ≈ 8 N, and Stiffness of *μ*_*S*,*K*_≈ 290 N/m; the subscript *α* identifies the stimulus type of Force A (*F*_*A*_), Force B (*F*_*B*_), or Stiffness (*K*). We characterized the ability of participants to perceive each of these standard signals, *μ*_*S*,*α*_, by selecting comparison signals, *μ*_*C*,*α*,*β*_ (for each stimulus type, *α*, and noise level, *β*), that were unique and that differed from the standard signals. The comparison signals were selected by using pilot testing to identify magnitudes at which participants were not always guessing and were not always correct when discriminating between the standard stimulus and comparison stimulus. Table 1 lists the mean magnitude of the haptically rendered standard and comparison stimuli across the five tested participants.

**Table 1:** Mean standard and comparison force and stiffness signals across the five participants.

	Noise Level, *β*	Stimulus Type, *α*		
		Force A (N)	Force B (N)	Stiffness (N/m)
Mean Standard Signal (Mean Comparison Signal)	None	5.1 (4.6)	7.6 (6.8)	250.9 (223.8)
	Low	5.5 (3.6)	8.2 (6.3)	302.2 (242.2)
	Medium	5.3 (3.2)	8.1 (6.1)	298.0 (217.8)
	High	5.2 (3.0)	8.0 (5.7)	293.5 (206.9)

A participant aimed to determine whether two presented stimuli were the same or different. The comparison signal was selected for each noise level and stimulus type to be a magnitude at which participants were not always guessing and were not always correct when discriminating between the standard stimulus and comparison stimulus. Mean standard and comparison signals reported are based on force sensor and encoder measurements (see ‘*Characterization of Haptic Rendering Quality*’ section below for more detail on how these signals were determined). Data are not included in this table for two participants who correctly identified all of the same trials at noise level *Low* for one stimulus type (Force A, Stiffness), three participants who correctly identified all of the different trials at noise level *Low* for one stimulus type (Force A, Force B), and one participant who correctly identified all of the different trials at noise level *Medium* for one stimulus type (Force A). For these six testing conditions, the empirically selected comparison signal was too different from the standard signal, and, in turn, we did not have enough sensitivity to identify the participant’s perceptual capabilities.

Force stimuli were rendered with the aim to attain and maintain a constant force. To transition from the stiff spring to the stimulus, the force initially ramped up from 0 N to a desired force, *F*_des_, where *F*_des_ = 3750 N/m · *x*_meas_ while *F*_meas_ < *F*_des_ − 0.3 N. The ramp up stiffness of 3750 N/m was selected to ensure that the linear spring behavior rendered for no more than 1/10 of the total penetration distance. The offset of 0.3 N was selected based on empirical testing to achieve a smooth transition from the rendering of an elastic spring to the rendering of a constant force; without this offset, the output force tended to overshoot *F*_des_ and the user could notice that the finger was being pulled back to the desired force. Once *F*_meas_ ≥ *F*_des_ − 0.3 N, the controller switched from rendering the linear spring to rendering a constant force; that is, the controller switched from rendering *F*_des_ = 3750 N/m · *x*_meas_ to rendering *F*_des_ for the remainder of the user’s pressing motion.

Stiffness stimuli were rendered with the aim of creating a linear spring. *F*_des_ was set as the desired stiffness, *k*_des_, multiplied by *x*_meas_.

We reiterate that the standard and comparison force and stiffness signals reported here are based on force sensor and encoder measurements. The desired signals, *F*_des_ and *k*_des_, are not reported to avoid confusion about the signals with which participants physically interacted.

### Controllers

We tested how adding haptically rendered noise to haptic force and stiffness stimuli impacted human perception of those stimuli. The purpose of the no-added-haptic-noise conditions was to identify the participant’s best ability to precisely detect the magnitude of the haptic stimulus; therefore, the haptic stimuli rendered were made as transparent as possible using a closed-loop (CL) controller. In contrast, the purpose of the added-haptic-noise conditions was to affect the participant’s ability to precisely detect the magnitude of the haptic stimulus by increasing the magnitude of the haptically rendered noise in the haptic stimulus. An open-loop controller was used for the noisy conditions rather than a closed-loop controller since a closed-loop controller could result in unstable behavior of the force-feedback device at the higher haptically rendered noise frequencies initially tested.

All force and stiffness stimuli were created by commanding that the following force be rendered at the location of interaction on the participant’s right index finger:

$$\begin{array}{c} F_{\text{cmd}}=\gamma F_{\text{des}}+k_{p}\left(F_{\text{des}}-{\hat{F}}_{\text{meas}}\right)+\text{NL}\cdot \text{HRN}, \end{array}$$ (1)

where *γ* and *k*_*p*_ are the respective dimensionless feedforward and feedback controller gains, ${\hat{F}}_{\text{meas}}$ is the real-time filtered finger force measurement, and NL is the dimensionless noise level, or scaling factor, of the haptically rendered noise, HRN. The term *γF*_des_ is a feed-forward term that aims to reduce the haptic rendering errors. The gain *k*_*p*_ works to make the actual force track the desired force and was set to 5 during the CL conditions. Furthermore, we reduce the instrumentation noise in *F*_meas_ to obtain ${\hat{F}}_{\text{meas}}$ using a first-order low-pass filter at the empirically selected cut-off frequency of 150 Hz. NL was set to null for the no-added-haptic-noise conditions, while *k*_*p*_ was set to null and NL was set to 5, 10, or 15 for the added-haptic-noise conditions of *Low*, *Medium*, and *High*. The haptic control loop and data storage executed at 1 kHz.

Decisions we considered when selecting our haptically rendered noise included the characteristics of the haptic noise (e.g., Gaussian versus non-Gaussian) and the manner by which to add the haptic noise to the haptic stimulus (e.g., as a function of time versus as a function of user position). In the end, HRN was empirically selected so that the magnitude of the haptic stimulus was not altered, the magnitude of the haptically rendered noise in the haptic signal increased, and the participant’s ability to identify the magnitude of the force and stiffness stimuli was affected. Additionally, HRN was designed to update as a function of time to ensure that the added haptically rendered noise was independent of the user’s movement.

**Fig 3:**
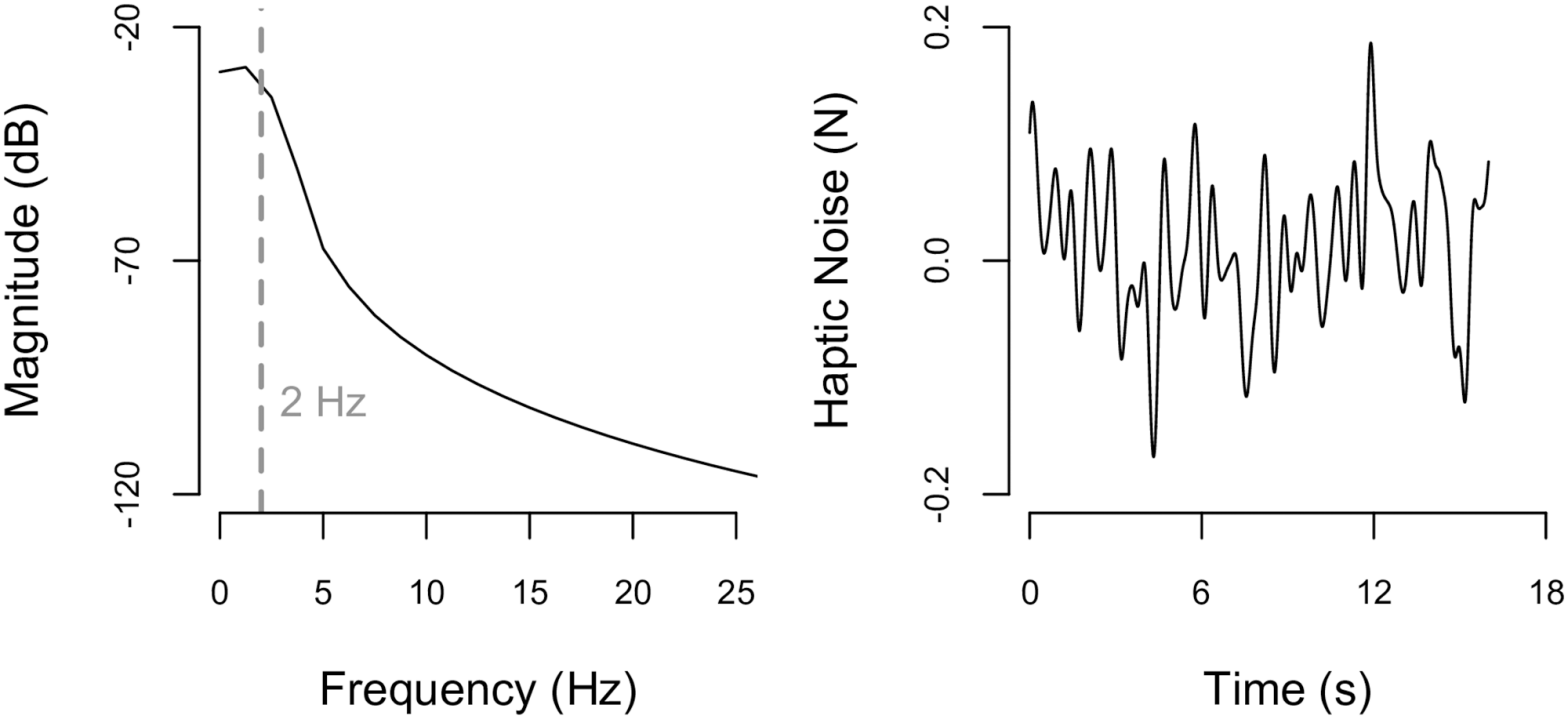
Haptically rendered noise. (Left) An example of the haptically rendered noise, HRN, power spectral density is given by the black solid line, and the low-pass filter cut-off frequency of 2 Hz is indicated by the gray dashed vertical line. The power spectral density was obtained using Welch’s averaged modified periodogram method of spectral estimation function in MathWorks’ Matlab (Massachusetts, United States), pwelch(). (Right) HRN is plotted as a function of time.

We initially selected HRN that was zero-mean white Gaussian, such that power was equally distributed across the range of input frequencies; however, the experimenter’s pilot testing demonstrated that her ability to precisely identify the magnitude of the force and stiffness stimuli was not impacted by this added HRN despite the fact that she could detect the haptically rendered noise (i.e., she could feel a high-frequency buzzing sensation). Her perception did deteriorate when the higher frequencies of the white Gaussian haptically rendered noise were removed while the haptically rendered noise’s amplitude was held constant, so HRN was designed as discretized zero-mean white Gaussian force noise that was filtered using a 2 Hz low-pass forward-backward fourth-order Butterworth filter. An example of the time and frequency response of HRN is given in Fig 3. The frequency response verifies that frequencies greater than 2 Hz indeed were attenuated. The standard deviation of this example HRN is 0.06 N.

**Fig 4:**
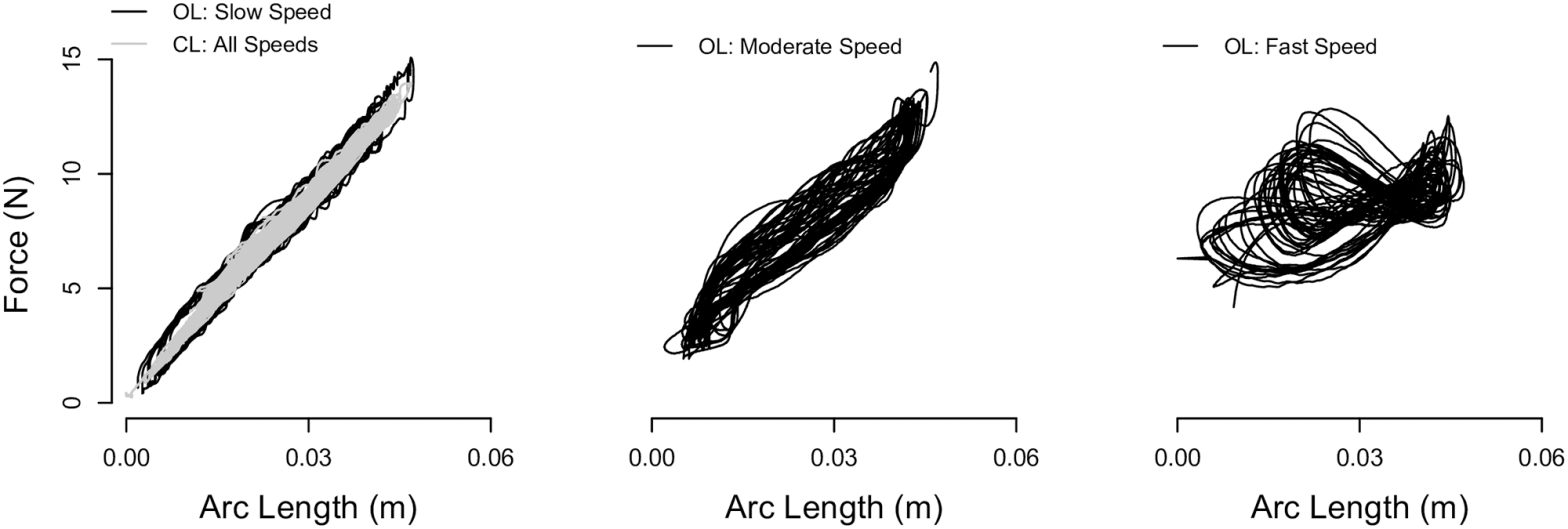
Sample interaction data. Example force versus arc length data when the force-feedback device rendered the standard Stiffness signal with no added haptically rendered noise using a closed-loop (CL) and open-loop (OL) controller and the experimenter pressed using slow, moderate, and fast interaction speeds.

We characterize the quality of the haptic stimuli for a given controller and user interaction speed, since limitations from the hardware and controllers of a force-feedback device can distort what a user feels [32, 33]. Data were collected when the experimenter rotated her finger in the OL and CL controlled device at speeds that were slow for 30 s, moderate for 20 s, and fast for 10 s while the standard Stiffness stimulus with no added haptically rendered noise was rendered. The device’s response to the experimenter’s movements is plotted in Fig 4. A power spectral density of the force obtained using Welch’s averaged modified periodogram method of spectral estimation identifies that the range of frequencies rendered span 0 to 5 Hz, corresponding to the frequencies at which humans control finger forces [11]. Furthermore, the interaction speeds were characterized by first smoothing the position data using smooth.spline in R with a smoothing parameter of *λ* = 0.9 (which modeled the data by an analytical function) and then differentiating the fitted function. The root-mean-square slow, moderate, and fast speeds, which identify 71% of the sine amplitude for each grouped velocity sinusoidal waveform, are 22.1°/s, 114.3°/s, and 209.2°/s, respectively, and the maximum slow, moderate, and fast speeds are 103.2°/s, 313.4°/s, and 486.2°/s, respectively. Ideal device rendering performance would give a highly linear force versus position response. Fig 4 visually portrays that the haptic rendering quality is most accurate during CL control across all speeds, while the haptic rendering quality deteriorates with an increase in the interaction speed during OL control.

### Characterization of haptic rendering quality

We developed a novel method by which we could characterize the magnitude of the haptic noise that also allowed us to compare the quality of rendering of haptic stimuli having differing dimensions (e.g., stimulus types of force and stiffness with the units of N versus N/m). After considering numerous approaches, including using the coefficient of determination and total least squares, we characterized the haptic noise as the magnitude of the force deviations with the common unit of N. In turn, we investigated how changes in the quality of the haptically rendered force and stiffness stimuli affect one’s ability to precisely identify the magnitude of these haptic stimuli.

We quantified the device’s rendering performance by first computing the magnitude of the force, *F*_mean_, and stiffness, *k*_mean_, stimuli as the mean of *F*_meas_i__ and $\frac{F_{{\text{meas}}_{i}}}{x_{{\text{meas}}_{i}}}$, respectively, where *i* are all analyzable data points. We then calculated the standard deviation of the haptic noise, or the standard deviation of the force data with respect to the mean force of the stimulus (expressed in units of N). The standard deviation of the force data for the stiffness stimuli is calculated using the force estimated from the mean stiffness, *F*_est_i__ = *k*_mean_ *x*_meas_*i*__, and the measured force, *F*_meas_i__. The standard deviation of the force data, or haptic noise, could then be compared across the force and stiffness testing conditions since they share the same dimension of N.

**Fig 5:**
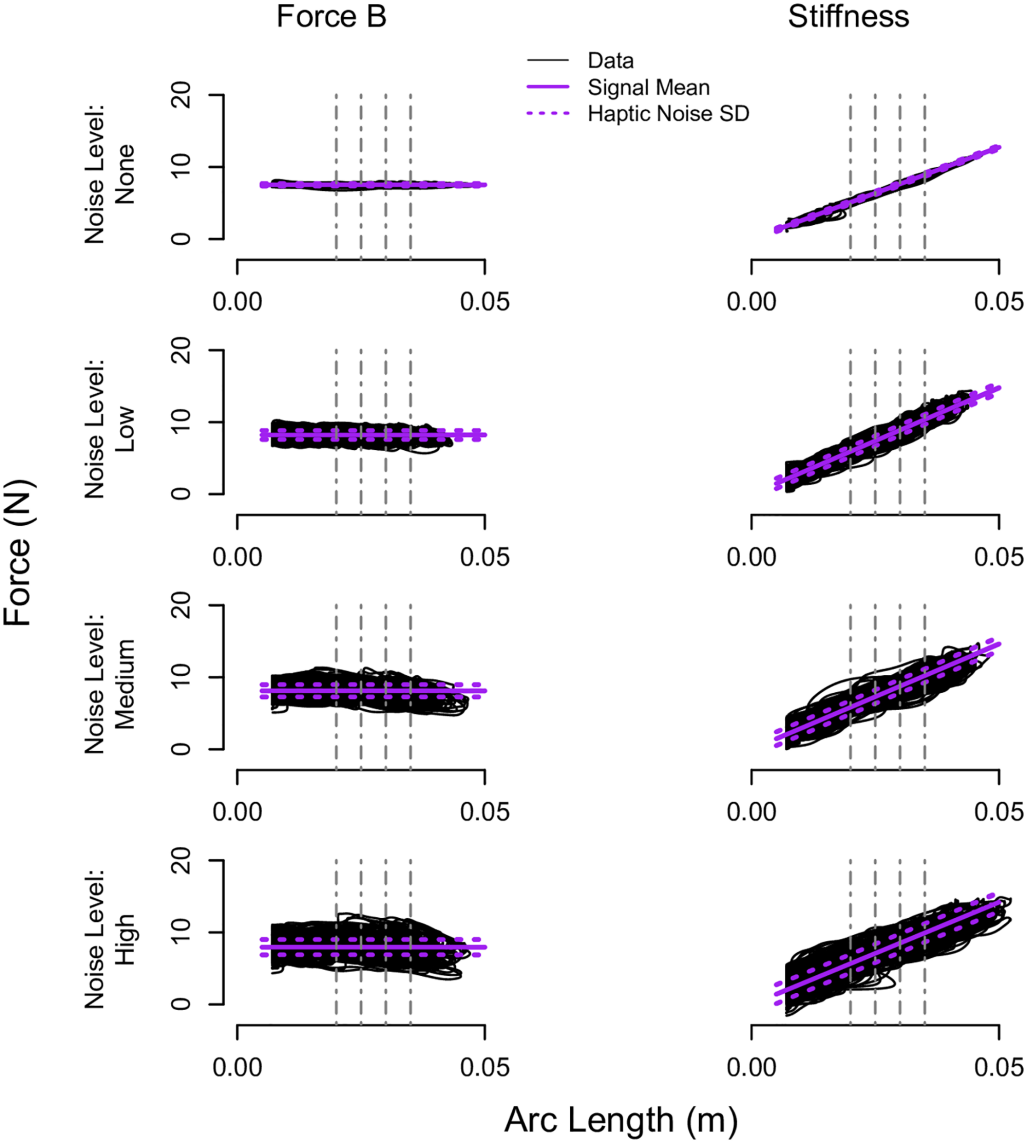
Representative participant data. Force versus arc length data for a representative participant when pressing on the standard Force B and Stiffness signals across each noise level (*None*, *Low*, *Medium*, and *High*). The smoothed and thresholded finger arc length data are indicated by the black solid lines, and the mean stimulus and the mean stimulus plus and minus the standard deviation of the haptic noise (Haptic Noise SD) are displayed by the purple solid and dotted lines, respectively. The commanded penetration arc lengths of P = 0.02, 0.025, 0.03, and 0.035 m are indicated by the vertical dashed and dotted gray lines.

Fig 5 plots representative data from when a participant pressed on the standard Force B and Stiffness signals across the four tested noise levels. The smoothed and thresholded finger arc length data, as discussed in the ‘*Data Analyses*’ section, are displayed by the black solid lines, and the magnitude of the haptic stimulus and the magnitude of the haptic stimulus plus and minus the standard deviation of the haptic noise are superimposed by the purple solid and dashed lines, respectively. As can be seen in Fig 5, the rendered stimuli line up well with the intended effects.

## Characterization of haptic perception capabilities

Below we discuss how we quantified each participant’s haptic sensitivity and response bias for every testing condition using the signal detection methods described in [30]. To characterize haptic sensitivity, the experimental methods seek to model each participant’s perception as a Gaussian probability distribution centered on a signal value, *μ*; uncertainty in the signal estimate dictates the curve’s standard deviation, *σ* [34]. Adopting assumptions outlined in Thurstone [1927], the Weber fraction, WF, combines these two measures to provide a single indicator of one’s ability to perceive:

$$\begin{array}{c} \text{WF}=\frac{\sqrt{2}\sigma }{\mu }, \end{array}$$ (2)

where a lower WF and *σ* indicate that an individual can more precisely identify a signal, *μ*. A benefit to characterizing human perception using the WF is that the value is dimensionless; thus, an individual’s perceptual performance can be compared across quantities with differing dimensions (e.g., N and N/m). Additionally, according to [6] the WF for force satisfies Weber’s law for forces between 2.5 N and 10.0 N.

The WF is obtained by first evaluating the dimensionless index of sensitivity, d′, for every condition and every participant [30]:

$$\begin{array}{c} d_{\alpha ,\beta }^{\prime }=2z\left(\frac{1+\sqrt{2p(c)-1}}{2}\right) \end{array}$$ (3)

where p(c) for an unbiased observer is:

$$\begin{array}{c} p\left(c\right)=\Phi \left(\frac{z\left(p_{H}\right)-z\left(p_{\text{FA}}\right)}{2}\right). \end{array}$$ (4)

*α* denotes the stimulus type, *β* denotes the noise level, *z*(·) denotes the z-score obtained using a quantile function with a standard normal distribution, and *p*_H_ and *p*_FA_ are the proportions of hits and false alarms. A hit occurs when a participant correctly indicates that the rendered haptic stimuli during a standard-standard trial are equal; the proportion of hits, *p*_*H*_, is *e*_*S*_, the number of same responses in the standard-standard trials, divided by *n*_*S*_, the total number of standard-standard trials. Similarly, a false alarm is when a participant incorrectly states that the rendered haptic stimuli during a standard-comparison trial are equal, as calculated from *e*_*C*_, the number of same responses in the standard-comparison trials, divided by *n*_*C*_, the total number of standard-comparison trials. Given that *n*_*S*_ = *n*_*C*_ = 72, we let *n* = *n*_*S*_ = *n*_*C*_. A larger d′ value indicates that one can more precisely identify the magnitude of a haptic stimulus. We used Table A5.3 in [30] to obtain the d′ values for this same-different experiment design.

The Weber fraction is then evaluated for every condition and every participant by:

$$\begin{array}{c} {\text{WF}}_{\alpha ,\beta }=\frac{\mu_{S_{\text{mean},\alpha }}-\mu_{C_{\text{mean},\alpha ,\beta }}}{d_{\alpha ,\beta }^{\prime }\cdot \mu_{S_{\text{mean},\alpha }}}. \end{array}$$ (5)

*μ*_*S*_mean,*α*__ and *μ*_*C*_mean,*α*,*β*__ are the mean values of the rendered standard and comparison haptic stimuli, respectively, for each stimulus type, *α*, and noise level, *β*, as evaluated in the ‘*Characterization of Haptic Rendering Quality*’ section; these values are given in the units of N or N/m for the force and stiffness stimulus types.

Last, we estimate the response bias, or criterion, c [30], for every condition and every participant as:

$$\begin{array}{c} c_{\alpha ,\beta }=-\left(\frac{z\left(p_{H,\alpha ,\beta }\right)+z\left(p_{\text{FA},\alpha ,\beta }\right)}{2}\right), \end{array}$$ (6)

where c = 0 indicates that the participant’s responses are not biased toward same or different judgements.

## Preparation of data analyses

Data were analyzed in *R*. Before we ran the analyses, the time-varying arc length, *x*_meas_, and force, *F*_meas_, data were smoothed by a smoothing spline, as discussed in the ‘*Controllers*’ section. Additionally, data were removed when the participant’s finger arc length was less than 0.007 m from *θ* = 0° (see Fig 1(c)) to eliminate the ramping up segment of the force stimulus (see the ‘*Magnitude of Haptic Stimuli*’ section), ensuring that only data from purposeful interactions with the haptic stimuli are analyzed. Furthermore, all trials were visually inspected to verify that two distinct pressing motions were extracted for the analysis. Data from ten of the 8,640 trials did not give two distinct pressing motions. Hence, for these ten trials the user interaction and haptic stimuli parameters were replaced with the mean values of the remaining trials for the corresponding participant, stimulus type, noise level, and comparison environment.

Next, we discuss how the data were analyzed. To begin, we report that the five participants who partook in this experiment discriminated stiffnesses comparably to another five participants in a separate, unpublished study; hence, we do not have reason to believe that the task performance of the five participants in this study was unusual. To determine whether the independent factors significantly affected the outcome measures in our study (as identified in the Results section), we fitted a linear mixed-effects model with repeated measures [35] using the function ‘lme’ in the *R* package ‘nlme’. Experimental conditions were treated as fixed effects, and participant was a random effect; interactions between the experimental condition levels were also investigated. Within-subject correlation between the experimental conditions’ levels was modeled using an AR(1) covariance structure [36] (function ‘update’ with correlation structure ‘corAR1()’). We identified significant effects using the function ‘anova’ in the package ‘lmerTest’ (using a Satterthwaite approximation for the degrees of freedom). We performed model selection in a hierarchical manner by first removing from each model non-significant interaction terms, followed by non-significant main effects. Finally, for factors with more than two levels, we constructed custom contrasts to determine which levels were significantly different from each other using the function ‘contrast’ in the package ‘lsmeans’. The normality assumption was checked by examining the distribution of the conditional residuals. As demonstrated by Oberfeld and Franke through simulations [37], linear mixed-effects models with sample sizes as small as five or six (with as many or fewer repeated measures) provide adequate control of the Type I error rate (i.e., finding an association when in fact none exists) when the data are normally distributed. Thus, we conclude that our significant findings are reliable despite the relatively small sample size of only five participants, since our data and model residuals were normally distributed, the fitted models were stable, and the Type I error rate was controlled.

## Results

The following results demonstrate that human perception of both suprathreshold haptic force stimuli and suprathreshold haptic stiffness stimuli is affected by the magnitude of the low-frequency haptic noise. Specifically, we show that participants perceived the magnitude of the haptic force and stiffness stimuli less precisely when low-frequency haptically rendered noise was added to the haptic stimuli, as well as when the magnitude of the haptic stimulus was smaller.

First, we confirm that participants interacted with the haptic device in a controlled and comparable manner. Next, we demonstrate that the quality of the haptic stimuli rendered was influenced by the added haptically rendered noise. Last, we verify that our method of adding low-frequency haptically rendered noise to the force and stiffness stimuli affected the ability of participants to precisely perceive the magnitude of suprathreshold force and stiffness stimuli.

### User interaction

**Fig 6:**
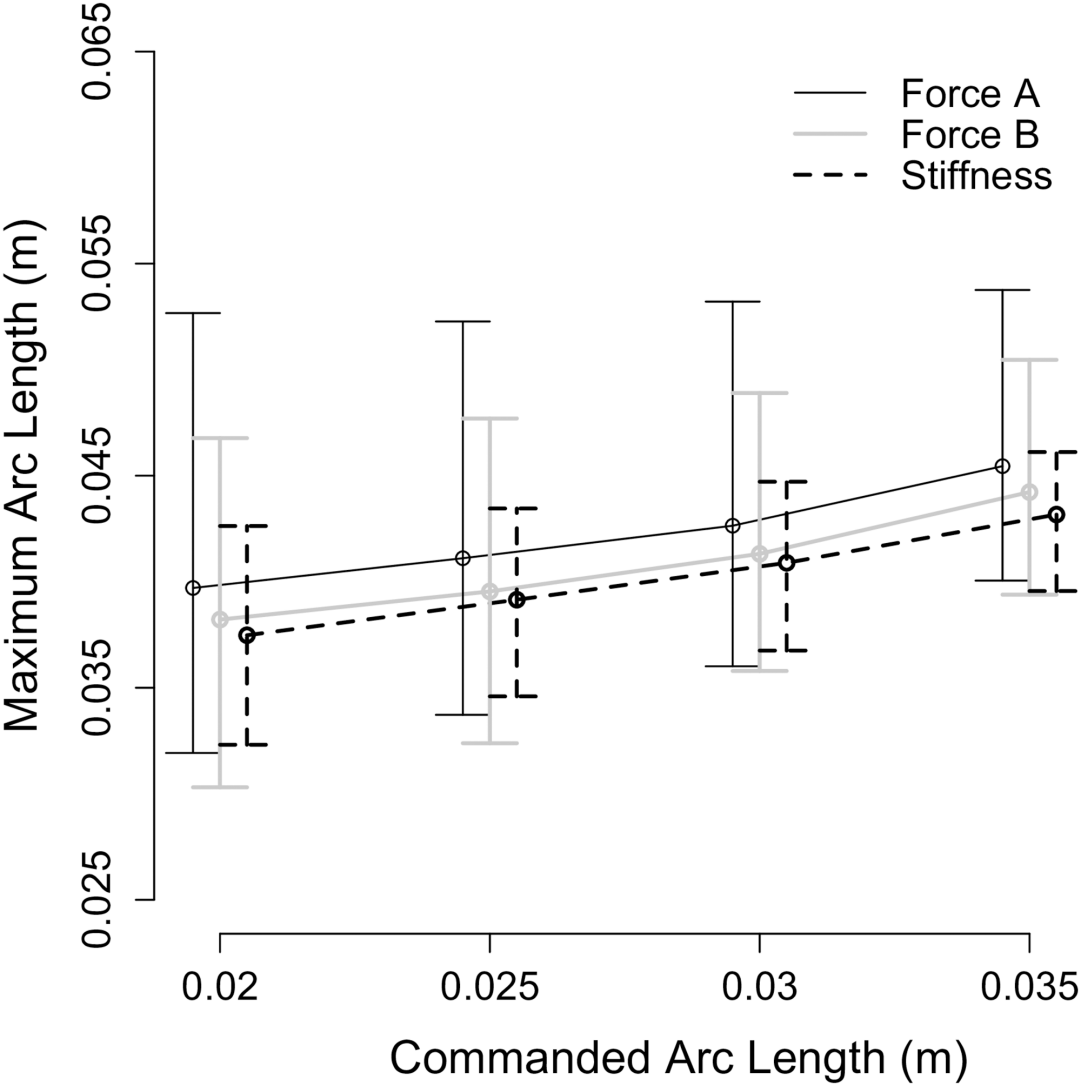
Maximum arc length to which participants’ fingers traveled. Median value (points) and lower and upper quartiles (error bars) of the maximum arc length to which participants’ fingers traveled across all trials for all participants are given as a function of the commanded arc length and stimulus type.

The experiment was designed so that the participant could not rely on terminal force cues to discriminate between the stiffness stimuli [6]; this goal was achieved by asking each participant to rotate his or her right index finger to a randomly commanded penetration arc length for every pressing motion. The maximum arc length to which a representative participant’s finger traveled is shown in Fig 5; the maximum arc lengths are visibly greater than the commanded penetration arc lengths (vertical dashed and dotted gray lines), because the participant began slowing the finger’s movement only when the finger reached P and the beep played. The maximum arc length to which the participant’s finger traveled is plotted as a function of the commanded arc length for each stimulus type in Fig 6.

We ran a three-way within subjects ANOVA with the factors of commanded penetration arc length, stimulus type, and noise level to determine the effect of these factors on the arc length that the participant’s finger traveled. The ANOVA confirmed that the maximum arc length to which the participant’s finger rotated was significantly impacted by the commanded penetration arc length (p<0.001), with significant differences between arc lengths of 0.030 m and 0.020 m (estimated mean difference ± standard error (EMD±SE): 0.003±0.001 m; p<0.001), 0.035 m and 0.020 m (EMD±SE: 0.005±0.001 m; p<0.001), and 0.035 m and 0.025 m (EMD±SE: 0.004±0.001 m; p < 0.001).

The experiment was designed to encourage the participant to interact with the haptic device using a pre-defined range of slow speeds, both to standardize interaction patterns across all trials and to avoid the rendering of imprecise haptic stimuli. Participants generally rotated the device using no faster than a moderate interaction speed, as defined in the ‘*Controllers*’ section, where the lower quartile, median, and upper quartile of the maximum speed across all trials was 113.2°/s, 149.1°/s, and 193.1°/s. A two-way within subjects ANOVA showed that the maximum participant interaction speed was not significantly impacted by the stimulus type (p = 0.459), noise level (p = 0.344), or their interaction (p = 0.109). Moreover, a two-way within subjects ANOVA also did not find a significant effect of stimulus type (p = 0.058), noise level (p = 0.414), or their interaction (p = 0.098) on the root-mean-square participant interaction speed.

The median time spent pressing on each haptic stimulus across all trials was 0.942 s, and the two-way within subjects ANOVA did not find the median time spent pressing on each haptic stimulus to be significantly impacted by the stimulus type, noise level, or their interaction.

### Haptic stimuli

**Fig 7:**
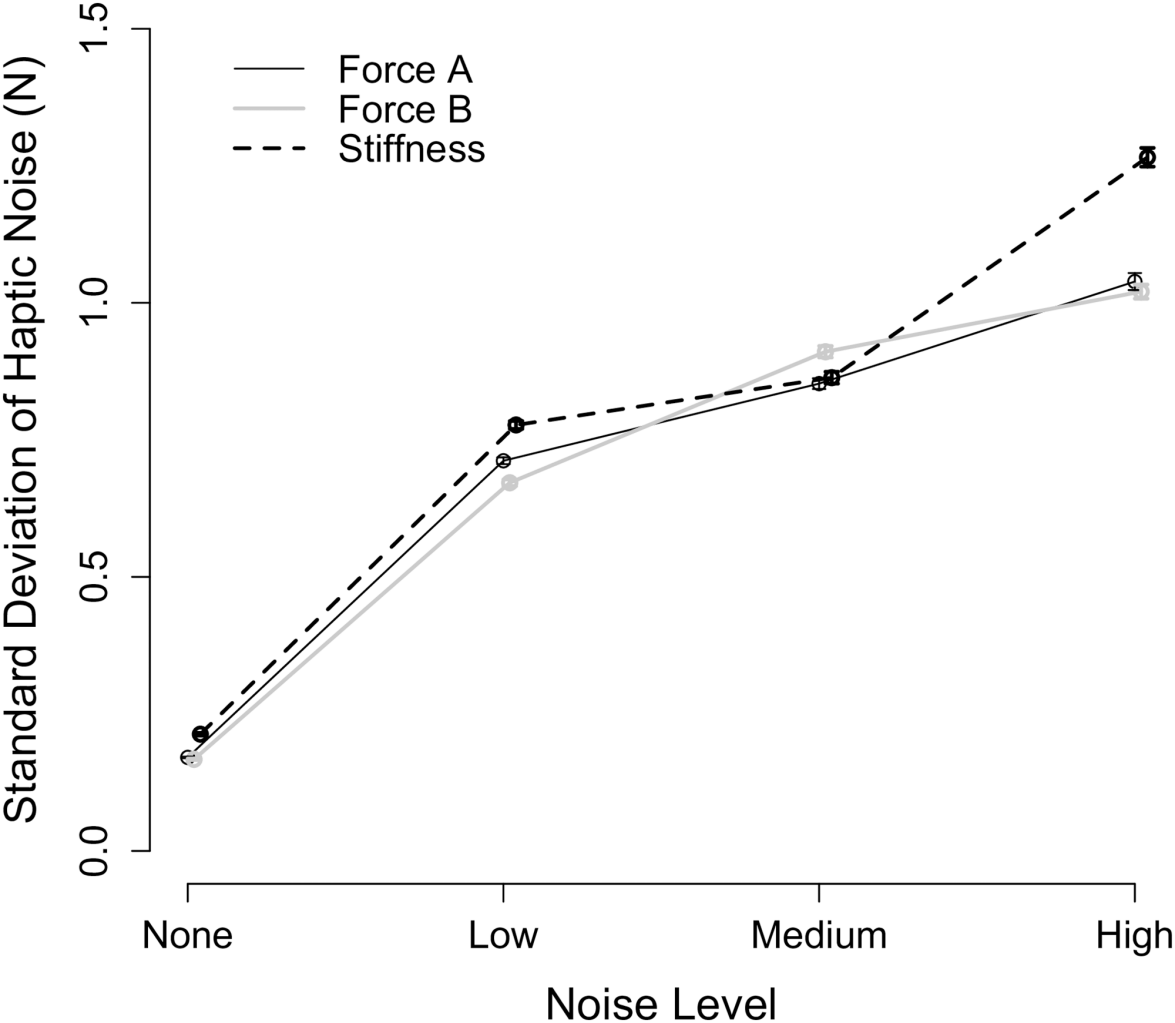
Haptic noise results. Mean value (points) plus and minus the standard error (error bars) of the standard deviation of the haptic noise across all trials for all participants is given as a function of the noise level and the stimulus type.

The haptic stimulus, as visually depicted in Fig 5, was precisely rendered when the noise level was *None*, while the haptic noise in the haptic stimulus increased with an increase in the noise level. The haptic rendering quality of the standard environment, as defined by the standard deviation of the haptic noise, is summarized across all tested conditions in Fig 7.

The added haptically rendered noise contributed, as desired, to the degradation of the haptic rendering quality. A two-way within subjects ANOVA found that the haptic rendering quality was significantly impacted by the stimulus type (p = 0.046) and the noise level (p<0.001), but not their interaction (p>0.05). The post-hoc pairwise comparisons did not find any significant differences between the grouped stimulus types (p = 0.999 for Force A and Force B; p = 0.081 for Force A and Stiffness; p = 0.076 for Force B and Stiffness). The mean standard deviation of the haptic noise across all stimulus types for noise levels *None*, *Low*, *Medium*, and *High* was 0.184 N, 0.720 N, 0.876 N, and 1.111 N, respectively, and it significantly differed for every combination of noise levels. The EMD±SE between the noise levels was −0.538±0.047 N for *None* and *Low* (p<0.001), −0.693±0.046 N for *None* and *Medium* (p<0.001), −0.926±0.046 N for *None* and *High* (p<0.001), −0.155±0.047 N for *Low* and *Medium* (p = 0.009), −0.388±0.046 N for *Low* and *High* (p<0.001), and −0.232±0.047 N for *Medium* and *High* (p<0.001).

These results also indicate that the device controller contributed to the degradation of the haptic rendering quality. The differences in the standard deviation of the haptic noise between noise levels *Low* and *Medium* and between *Medium* and *High* were on the order of 0.15 N and 0.23 N, when only the magnitude of the added haptically rendered noise changed. The greatest change in the standard deviation of the haptic noise occurred between noise levels *None* and *Low* (on the order of 0.54 N), when the controller changed from CL to OL control and haptically rendered noise was purposefully added.

We conclude that, aligned with the experimental aims, the quality of the rendered haptic stimuli deteriorated with an increase in the noise level. Additionally, we underscore that our method for characterizing haptic noise allows for a comparison of haptic rendering quality in haptic stimuli with differing dimensions (e.g., N versus N/m).

### Haptic perception

Below we characterize the sensitivity of our participants to each stimulus type and each noise level. Additionally, we present the participants’ response biases for each of the twelve testing conditions (three stimulus types × four noise levels).

#### Participant responses

First, we assessed whether the comparison stimuli selected were appropriate for characterizing participant perceptual capabilities. We identified the percentage of participant correct responses for each tested condition, where a percentage of participant correct responses of 50% indicates that the participant was always guessing (comparison signal was too similar to the standard signal) and 100% indicates that the participant could easily distinguish between the two haptic stimuli (comparison signal was too different from the standard signal). The percentage of participant correct responses corresponds to the total number of responses when the participant indicated correctly that the environments were the same or were different, divided by the total number of trials (and multiplied by 100).

**Fig 8:**
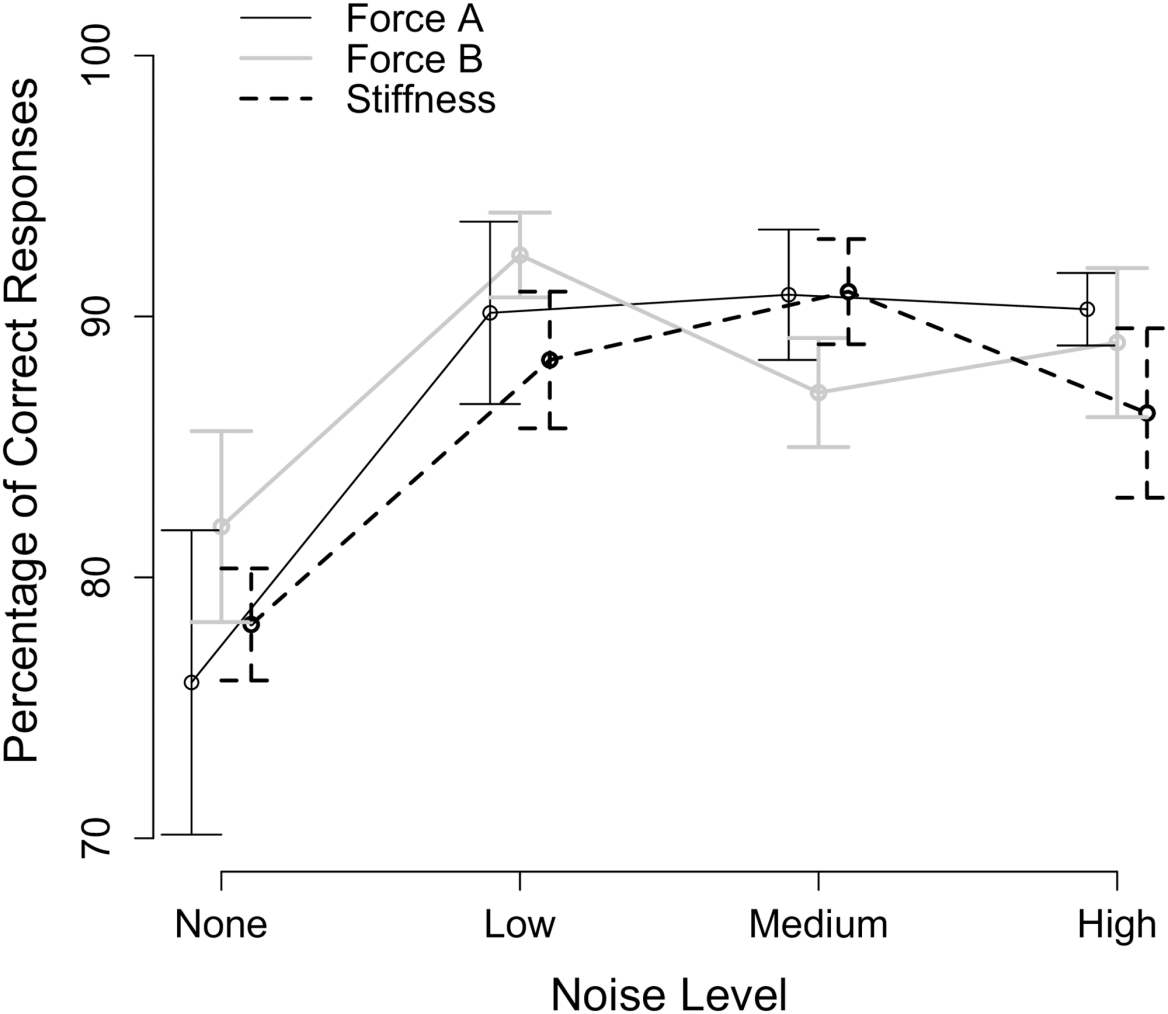
Percentage of participant correct responses. Mean value (points) plus and minus the standard error (error bars) of the percentage of participant correct responses across all participants are given as a function of the noise level and the stimulus type.

The percentages of correct responses for all participants are summarized in Fig 8 and demonstrate that the selected comparison signals presented a reasonable challenge: the participants were not always guessing and were not always correct. The mean percentage of participant correct responses for noise levels *None*, *Low*, *Medium*, and *High* was 78.7%, 90.3%, 89.6%, and 88.5%, respectively. Upon further inspection of the percentage of correct responses, we found that two participants correctly identified all of the same trials (*p*_H_ = 1) at noise level *Low* for one stimulus type (Force A, Stiffness), three participants correctly identified all of the different trials (*p*_FA_ = 1) at noise level *Low* for one stimulus type (Force A, Force B), and one participant correctly identified all of the different trials (*p*_FA_ = 1) at noise level *Medium* for one stimulus type (Force A). For these six testing conditions, the empirically selected comparison signal was too different from the standard signal, and, in turn, we did not have enough sensitivity to identify the participant’s perceptual capabilities (i.e., the z-score and, in turn, the index of sensitivity approached infinity; the WF approached 0). Therefore, we replaced d′, WF, and c for these six testing conditions with the corresponding mean value using the remaining participants at the same noise level and stimulus type.

#### Haptic sensitivity

**Fig 9:**
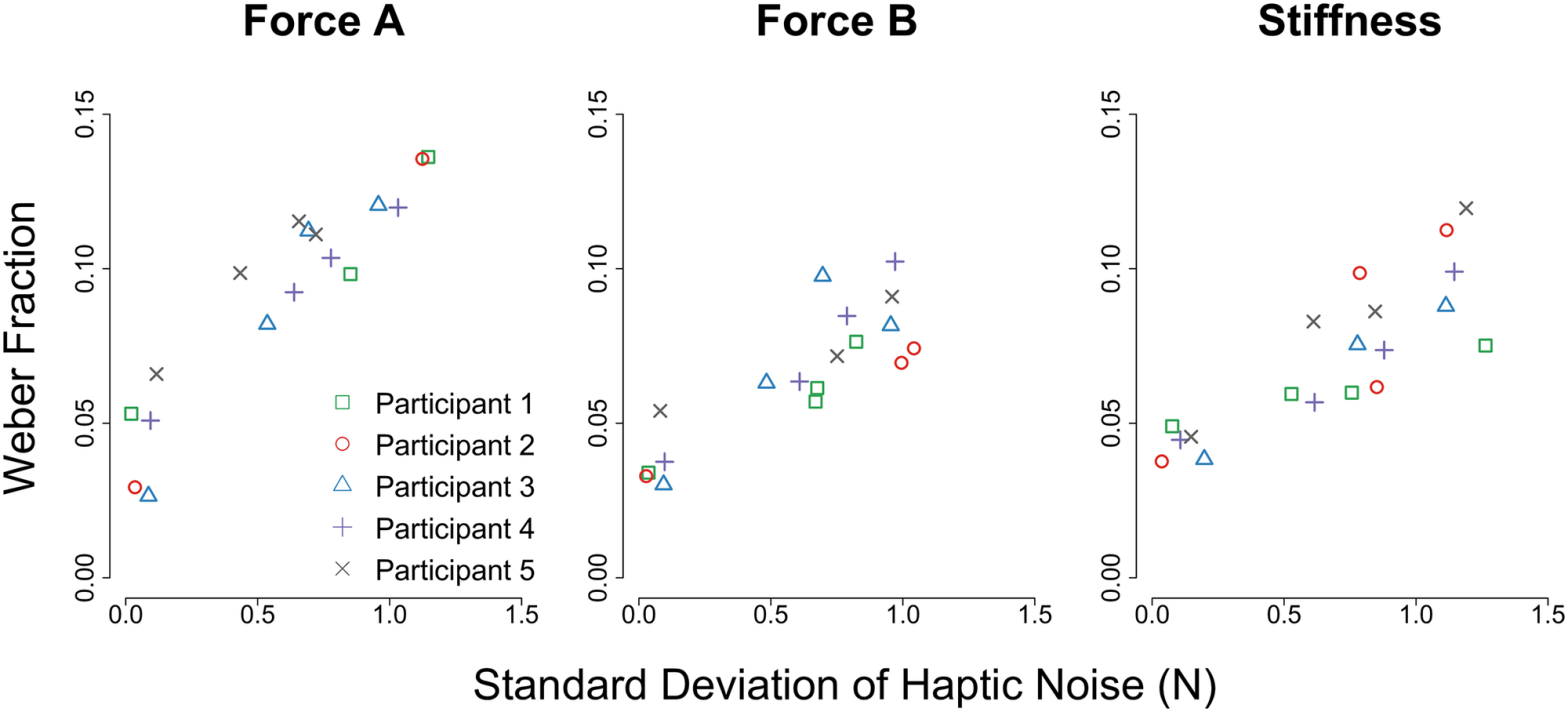
Weber fraction results—Raw data. The Weber fraction is given as a function of the mean standard deviation of the haptic noise for each participant and noise level during the (Left) Force A, (Middle) Force B, and (Right) Stiffness conditions.

Below we demonstrate that adding low-frequency haptically rendered noise to our haptic stimuli affected participants’ abilities to perceive the haptic stimuli. Participants’ perceptual capabilities were characterized for each stimulus type and noise level by the WF, and results are summarized in Fig 9.

First, we found that an individual’s ability to perceive the haptic stimuli is greatly related to the standard deviation of the added haptic noise. The Pearson product-moment correlation coefficient indicates a strong linear correlation between the standard deviation of the haptic noise and the Weber fraction, with *ρ* = 0.94, *ρ* = 0.85, and *ρ* = 0.84 for the Force A, Force B, and Stiffness testing conditions, respectively.

Next, we assessed whether Weber’s law held; that is, we investigated whether the force WF was independent of the nominal force of ∼5 N and ∼8 N. The mean WF for Force A (∼5 N) and Force B (∼8 N) at the noise level *None* was 0.045 and 0.038, respectively. A Welch two sample t-test was run on the noise level *None* results for our Force A and Force B testing conditions. A significant difference in WFs was not found between these two force magnitudes (*t*(6.32) = 0.859, *p* = 0.422) suggesting that the WF did not depend on the magnitude of the nominal force for the tested force magnitudes.

**Fig 10:**
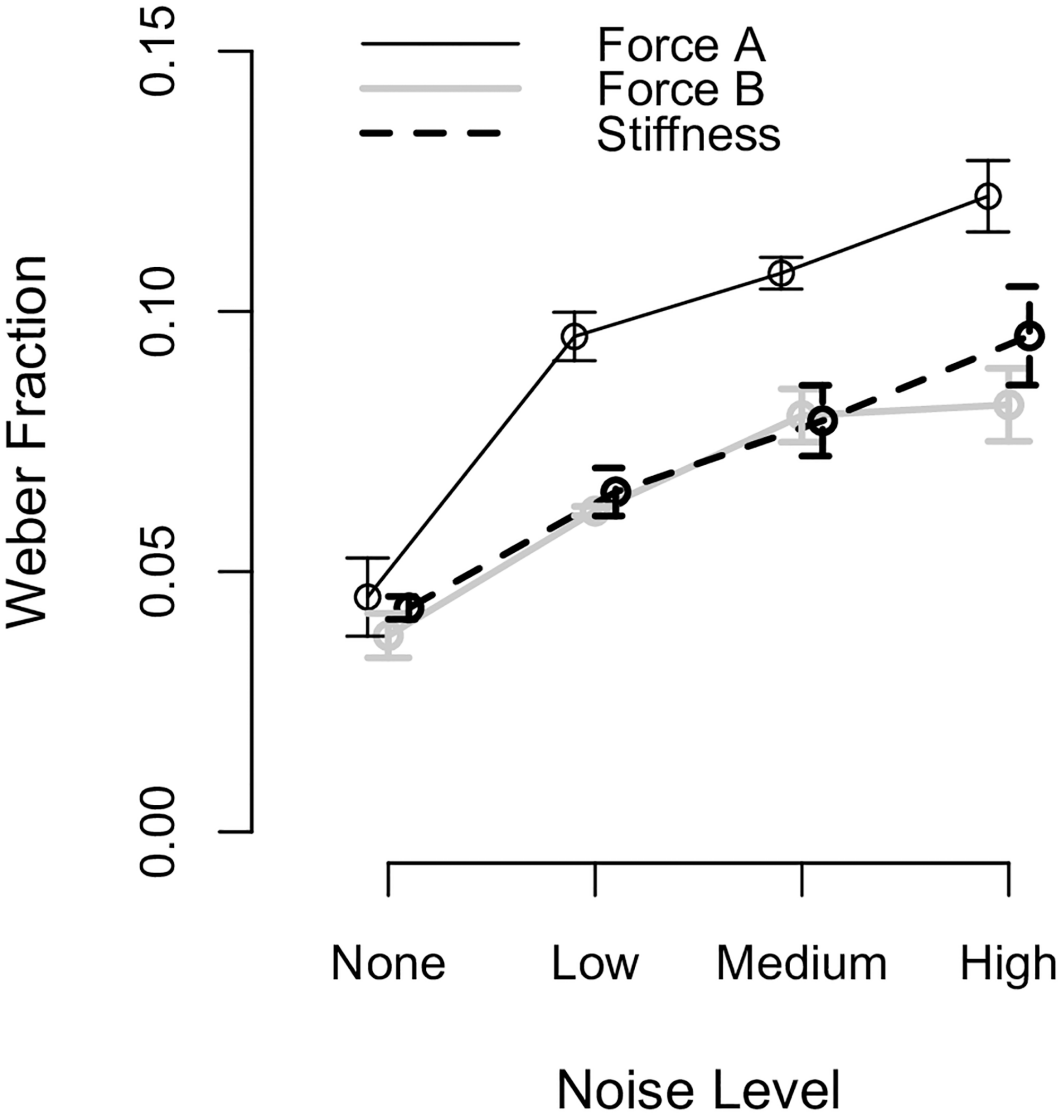
Weber fraction results. Mean value (points) plus and minus the standard error (error bars) of the Weber fraction across all participants is given as a function of the stimulus type and the noise level.

We continued our analyses by showing that the ability of participants to precisely identify the magnitude of the force and stiffness stimuli across all testing conditions, as characterized by the WF, was influenced by both the magnitude of the haptically rendered noise and the magnitude of the haptic stimulus (see Fig 10). A two-way within subjects ANOVA revealed that the WF was significantly affected by the main effects of stimulus type (p<0.001) and noise level (p<0.001), as well as their interaction (p = 0.027).

Participants could better identify the larger force magnitude of Force B than the smaller force magnitude of Force A when the same magnitude of haptically rendered noise was added to each stimulus. The mean WF values of Force A, Force B, and Stiffness were 0.092, 0.065, and 0.071, respectively. Force A gave significantly larger WFs than Force B at noise levels *Low* (EMD±SE: 0.034±0.007; p<0.001), *Medium* (EMD±SE: 0.027±0.007; p = 0.002), and *High* (EMD±SE: 0.040±0.007; p<0.001). Additionally, Force A gave significantly larger WFs than Stiffness at noise levels *Low* (EMD±SE: 0.030±0.008; p<0.001), *Medium* (EMD±SE: 0.029±0.008; p = 0.001), and *High* (EMD±SE: 0.023±0.008; p<0.010).

Participants could identify the magnitude of the haptic stimulus better when the magnitude of the added haptically rendered noise was smaller. The mean WF values across all stimulus types for noise levels *None*, *Low*, *Medium*, and *High* were 0.042, 0.074, 0.089, and 0.101, respectively. For Force A, the WF was significantly lower for noise level *None* than *Low* (EMD±SE: −0.050±0.007; p<0.001), *None* than *Medium* (EMD±SE: −0.062±0.007; p<0.001), *None* than *High* (EMD±SE: −0.077±0.007; p<0.001), and *Low* than *High* (EMD±SE: −0.027±0.007; p = 0.004). For Force B, the WF was significantly lower for noise level *None* than *Low* (EMD±SE: −0.023±0.007; p = 0.014), *None* than *Medium* (EMD±SE: −0.042±0.007; p<0.001), *None* than *High* (EMD±SE: −0.044±0.007; p<0.001), and *Low* than *High* (EMD±SE: −0.021±0.007; p = 0.033). For Stiffness, the WF was significantly lower for noise level *None* than *Low* (EMD±SE: −0.022±0.007; p = 0.021), *None* than *Medium* (EMD±SE: −0.036±0.007; p<0.001), *None* than *High* (EMD±SE: −0.056±0.007; p<0.001), *Low* than *High* (EMD±SE: −0.034±0.007; p<0.001), and *Medium* than *High* (EMD±SE: −0.020±0.007; p = 0.043). Given that the participants’ perceptual results for noise levels *Low* and *High* significantly differed for all three stimulus types, we conclude that perceptual deterioration occurred due to the added low-frequency haptically rendered noise (the OL controller was employed for both of these noise levels).

Participants’ perceptual abilities may also have been impacted by the noise arising due to the participant moving in an OL versus a CL controlled environment (see ‘*Controllers*’ section). The difference in the standard deviation of the haptic noise was much greater between noise levels *None* and *Low* than between noise levels *Low* and *Medium* and noise levels *Medium* and *High*. The large difference in the magnitude of the haptic noise between noise levels *None* and *Low* arose in part due to the change from using a CL controller to an OL controller. We propose that participant perceptual performance may be impacted by the haptic noise arising when switching from using a CL controller to using an OL controller, since participant perceptual performance significantly differed between noise levels *None* and *Low* (yet not between noise levels *Low* and *Medium* for all stimulus types and not between noise levels *Medium* and *High* for stimulus types Force A and Force B).

The main finding of these perceptual results is that our added low-frequency haptically rendered noise, as well as the magnitude of the haptic stimulus, affected participants’ abilities to precisely identify the magnitude of the force and stiffness stimuli. We thus conclude that an individual’s ability to identify suprathreshold haptic force and stiffness stimuli is impacted by the magnitude of the low-frequency haptic noise in the stimulus.

#### Response bias

We examine here whether participants reported more often that the two haptic stimuli presented during a trial were the same or different (i.e., response bias, c) for the differing testing conditions. A two-way within subjects ANOVA revealed that participants’ responses as to whether the environments were the same or different were influenced by both the stimulus type (p = 0.002) and the noise level (p = 0.006). The criterion for Stiffness was significantly greater than the criterion for Force A (EMD±SE: −0.277 ± 0.081; p = 0.003) and Force B (EMD±SE: −0.236 ± 0.078; p = 0.011), and the criterion for noise level *Low* was significantly greater than the criterion for noise level *None* (EMD±SE: −0.473 ± 0.142; p = 0.009). Therefore, the criterion that participants used to decide whether the environments were the same was affected by the type of environment with which they interacted.

## Discussion

This work lays the foundation for future research to investigate how human perception of force relates to human perception of stiffness. This study aimed to identify a method by which an individual’s ability to identify suprathreshold haptic stiffness stimuli, as well as suprathreshold haptic force stimuli, can be degraded through the addition of haptic force noise. Prior work has demonstrated that the ability of an individual to detect suprathreshold haptic force stimuli can be degraded by the addition of suprathreshold haptic force noise to the stimuli. Here, the main finding is that low-frequency haptically rendered noise affected participants’ abilities to perceive both haptic stiffness stimuli and haptic force stimuli. Additionally, our results demonstrate that participant perception of haptic stimuli is affected by both the magnitude of the haptic stimulus and the magnitude of the haptic noise. Below we compare the results of our study to the results found in the literature, propose mechanoreceptors that may have contributed to participants’ altered force and stiffness perceptual abilities, and indicate research areas that could benefit from the findings obtained in this work.

First, we compare our results in the absence of added haptically rendered noise to the results found in the literature. As seen in Fig 10, the WF for both force and stiffness perception in the no-added-haptic-noise conditions was 0.04. Other groups previously obtained WFs on the order of 0.10 [38] and on the order of 0.20 [39] when participants perceived forces by pressing with the index finger. Additionally, force and stiffness WFs were on average 0.14 and 0.22, respectively, in [6], while stiffness WFs were on average 0.11 in [40]. Reasons for our lower WF values include that our testing methods, haptic stimuli, and experimental setups differed from those used in prior work. For example, we permitted the participant to press only once on each haptic stimulus in order to control the number of interactions (and, in turn, the information available to the participant about each haptic stimulus). Allowing the participant to press each haptic stimulus multiple times might have led to different participant performance (i.e., higher or lower WFs). Additionally, this study might have obtained estimates of participants’ Weber fractions that better match existing literature if a two-alternative force-choice task had been used rather than a same-different task.

Even so, our results demonstrate that participant perception of suprathreshold force and stiffness stimuli was affected by the presence of low-frequency haptic noise. That is, participant perceptual capabilities (i.e., WF) were strongly linearly correlated with the quality of the haptic rendering (i.e., standard deviation of haptic noise). Given that the addition of the low-frequency haptically rendered noise to the haptic stimulus affected the ability of participants to perceive the magnitude of both haptic force and stiffness stimuli, we conclude that the mechanoreceptors contributing to both force and stiffness perception were providing less precise information. Golgi tendon organs and SA I units are the mechanoreceptors that most likely contributed to our observed perceptual results, as they are sensitive to low-frequency force stimulation [14, 15].

We suggest that the Golgi tendon organs contributed to participants’ impaired abilities to precisely detect the magnitude of the haptic stimuli in the presence of the haptic noise. Golgi tendon organs respond to the force magnitudes used in this study (i.e., 1 to 18 N) and have a dynamic range that overlaps with the frequency content of our added force noise [16, 41, 42]. Additionally, Golgi tendon organs are sensitive to active muscle contraction and not to passive stretching [15]; our participants needed to contract their muscles to flex and rotate their fingers about the MCP joint, so the force information detected by the Golgi tendon organs was likely affected by the haptic noise.

We also propose that SA I units may have contributed to the participants’ impaired ability to precisely detect the magnitude of the haptic stimuli. To the best of our knowledge, prior research has not identified how SA I afferents respond to low-frequency fluctuations of forces between 1 to 18 N. One study showed that SA I afferents in the glabrous skin of adults’ hands are most responsive to forces <1 N and rapidly level off thereafter [14], while another study found that six out of ten SA I afferents from the foot and tibial part of the human leg plateau before forces reach 7 N [43]. While the SA I afferents at the location of interaction during the force perception trials may have been saturated [14, 43], SA I afferents located to the periphery of the interaction region that were not saturated may have responded to the fluctuating forces.

Future work could determine which neural mechanism(s) are contributing to an individual’s altered perceptual performance in the presence of low-frequency haptic noise. For example, studies could more definitively determine whether the kinesthetic (i.e., Golgi tendon organs) or cutaneous (i.e., SA I units) mechanoreceptors affect the human ability to identify noisy force and stiffness stimuli by applying a local anesthetic agent to the skin of the finger and then quantifying one’s ability to perceive the noisy stimuli based on only the kinesthetic contributors. Recent work also proposes that muscle spindles may contribute to force perception [44, 45]; therefore, further research could investigate the plausibility of the muscle spindles contributing to the altered perception, in addition to the aforementioned Golgi tendon organs or SA I units. Last, further research could investigate the bandwidth and corresponding magnitudes of sinusoidal haptic noise stimulation waveforms that impact a human’s ability to detect force and stiffness signals to determine the effect of various amplitudes of low- and high-frequency stimuli on human perception.

Knowledge about how haptic noise affects an individual’s ability to perceive haptic force stimuli and haptic stiffness stimuli can benefit a number of research areas. An understanding of how humans perceive haptic stimuli in the absence and presence of haptic noise can aid researchers in the development of robust models that relate how an individual perceives force and stiffness stimuli. Additionally, information about how differing frequency content affects an individual’s ability to perceive force and stiffness stimuli can be used in the design of haptic watermarks [24], so that haptic watermarks have little to no impact on human perception. Finally, the functionality of peripheral pathways may be investigated in individuals with haptic perceptual impairments by identifying whether an individual can detect changes in a haptic stimulus when haptic noise is added; an ability to identify a change in the haptic stimulus would indicate that peripheral pathways are still functioning.

This study contributes to a larger body of work that aims to model how humans perceive stiffness. We demonstrated that mechanically stimulating the right index finger’s distal pad with low-frequency haptic noise affects a human’s ability to perceive both force and stiffness stimuli. In turn, we provided a plausible explanation for why the Golgi tendon organs and SA I units may contribute to the altered force and stiffness percepts. Future research could apply the aforementioned experimental methods to further explore the processes governing a human’s perception of force and stiffness, with possible application to areas including haptic watermarking and assessment of the functionality of peripheral pathways in individuals with haptic impairments.
